# Spread of COVID-19 Vaccine Misinformation in the Ninth Inning: Retrospective Observational Infodemic Study

**DOI:** 10.2196/33587

**Published:** 2022-03-16

**Authors:** Alec J Calac, Michael R Haupt, Zhuoran Li, Tim Mackey

**Affiliations:** 1 School of Medicine University of California San Diego San Diego, CA United States; 2 Global Health Policy and Data Institute San Diego, CA United States; 3 Department of Cognitive Science University of California San Diego San Diego, CA United States; 4 S-3 Research San Diego, CA United States; 5 Rady School of Management University of California San Diego San Diego, CA United States; 6 Global Health Program Department of Anthropology University of California San Diego La Jolla, CA United States

**Keywords:** infoveillance, infodemiology, COVID-19, vaccine, Twitter, social listening, social media, misinformation, spread, observational, hesitancy, communication, discourse

## Abstract

**Background:**

Shortly after Pfizer and Moderna received emergency use authorizations from the Food and Drug Administration, there were increased reports of COVID-19 vaccine-related deaths in the Vaccine Adverse Event Reporting System (VAERS). In January 2021, Major League Baseball legend and Hall of Famer, Hank Aaron, passed away at the age of 86 years from natural causes, just 2 weeks after he received the COVID-19 vaccine. Antivaccination groups attempted to link his death to the Moderna vaccine, similar to other attempts misrepresenting data from the VAERS to spread COVID-19 misinformation.

**Objective:**

This study assessed the spread of misinformation linked to erroneous claims about Hank Aaron’s death on Twitter and then characterized different vaccine misinformation and hesitancy themes generated from users who interacted with this misinformation discourse.

**Methods:**

An initial sample of tweets from January 31, 2021, to February 6, 2021, was queried from the Twitter Search Application Programming Interface using the keywords “Hank Aaron” and “vaccine.” The sample was manually annotated for misinformation, reporting or news media, and public reaction. Nonmedia user accounts were also classified if they were verified by Twitter. A second sample of tweets, representing direct comments or retweets to misinformation-labeled content, was also collected. User sentiment toward misinformation, positive (agree) or negative (disagree), was recorded. The Strategic Advisory Group of Experts Vaccine Hesitancy Matrix from the World Health Organization was used to code the second sample of tweets for factors influencing vaccine confidence.

**Results:**

A total of 436 tweets were initially sampled from the Twitter Search Application Programming Interface. Misinformation was the most prominent content type (n=244, 56%) detected, followed by public reaction (n=122, 28%) and media reporting (n=69, 16%). No misinformation-related content reviewed was labeled as misleading by Twitter at the time of the study. An additional 1243 comments on misinformation-labeled tweets from 973 unique users were also collected, with 779 comments deemed relevant to study aims. Most of these comments expressed positive sentiment (n=612, 78.6%) to misinformation and did not refute it. Based on the World Health Organization Strategic Advisory Group of Experts framework, the most common vaccine hesitancy theme was individual or group influences (n=508, 65%), followed by vaccine or vaccination-specific influences (n=110, 14%) and contextual influences (n=93, 12%). Common misinformation themes observed included linking the death of Hank Aaron to “suspicious” elderly deaths following vaccination, claims about vaccines being used for depopulation, death panels, federal officials targeting Black Americans, and misinterpretation of VAERS reports. Four users engaging with or posting misinformation were verified on Twitter at the time of data collection.

**Conclusions:**

Our study found that the death of a high-profile ethnic minority celebrity led to the spread of misinformation on Twitter. This misinformation directly challenged the safety and effectiveness of COVID-19 vaccines at a time when ensuring vaccine coverage among minority populations was paramount. Misinformation targeted at minority groups and echoed by other verified Twitter users has the potential to generate unwarranted vaccine hesitancy at the expense of people such as Hank Aaron who sought to promote public health and community immunity.

## Introduction

On January 5, 2021, Major League Baseball (MLB) legend and Hall of Famer, Hank Aaron, publicly received his first dose of the Moderna vaccine series at Morehouse School of Medicine in Atlanta, Georgia, USA. Two weeks later, he passed away at the age of 86 years due to natural causes. Following his death, a prominent antivaccine activist and founder of a known antivaccine group posted information on the popular microblogging site Twitter, which claimed an unfounded link between Aaron’s death and the COVID-19 vaccine [1]. This claim was erroneously based on reports of elderly deaths following COVID-19 vaccination reported into the Vaccine Adverse Event Reporting System (VAERS). VAERS, established in 1990, is a public US database and passive reporting system comanaged by the US Centers for Disease Control and Prevention and the Food and Drug Administration, where individuals can submit vaccine adverse event reports without clinical verification. Data provided by VAERS has been increasingly used by antivaccine advocates to spread misinformation [2]. This database has also seen increased reports since COVID-19 vaccines received emergency use authorization from the Food and Drug Administration [2].

In this retrospective observational event-driven infoveillance study, we sought to characterize public user reaction to the death of Hank Aaron on Twitter for purposes of expanding the literature on minority-specific COVID-19 misinformation topics as detected on social media platforms. Twitter was chosen for this study because previous research has shown that misinformation, and specifically vaccine-related misinformation and disinformation (including general antivaccination topics, misinformation about non–COVID-19 vaccines, and misinformation specific to COVID-19 vaccines), is prominent on the platform [3-8]. Additionally, information and news reports about both Aaron’s initial vaccination and his death were shared on Twitter making it an ideal platform to further explore user interaction, sentiment, and dissemination of this content.

For example, a study conducted in 2021, which followed approximately 138 million tweets from a set of specific antivaccine-related keywords and accounts known to post antivaccine narratives detected large thematic clusters containing debunked claims and conspiracies along with misinformation originating from noncredible sources [7]. Another study, which examined 1.8 million vaccine-related tweets collected from 2014 to 2017, used topic modeling to identify 22% of their data set as containing antivaccine sentiment [5]. In response to growing concerns and studies identifying misinformation content, Twitter changed its content moderation policies during the COVID-19 pandemic, including, but not limited to, applying labels to tweets that contain vaccine misinformation, removing misleading content deemed to be harmful to the public, and suspending user accounts for posting COVID-19 and vaccine-related misinformation on a 5-strike system [8-10].

Hence, the primary objective of this study was to identify and characterize the impact of a specific event and assess whether it generated different types of vaccine-related misinformation. We also sought to understand the types of users who disseminated and amplified this misinformation, the overall sentiment of users toward vaccines, and if this social media-based dissemination influenced other online users’ attitudes and beliefs about COVID-19 vaccine hesitancy and confidence. A subanalysis of this study also focused on whether specific online minority user populations and verified Twitter accounts were also active and engaged in this misinformation discourse.

## Methods

### Data Collection

The Twitter Search Application Programming Interface (API) allows for the retrospective collection and return of a collection of relevant tweets meeting a specific search criterion (such as certain keywords, hashtags, or account handles) within a specific period. We used the v1.1 Twitter Search API, which allowed for simple queries against the indices of recent and popular tweets up to a maximum of 7 days and behaved similarly to, but not exactly like the Search User Interface feature available in Twitter mobile and web clients [11]. Tweets and user comments associated with the keywords “Hank Aaron” and “vaccine” were queried with the Twitter Search API from January 31, 2021, to February 6, 2021 (the week immediately following the death of Hank Aaron), hereinafter referred to as the “Initial Sample.” This time frame was chosen based on previous literature that has shown that Twitter users generally have a circaseptan (7 day) pattern for negative and positive sentiment in response to information on Twitter, with other similar COVID-19 misinformation research on the platform using similar data collection time frames [12,13]. From tweets collected in the Initial Sample, the authors manually coded for tweets relevant to vaccine misinformation (as detailed in the “Theoretical Framework” and “Content Coding” sections below) and then identified an additional set of Twitter direct user comments that interacted with these tweets for further analysis, hereinafter referred to as “Seeded Sample.” Data collection was not limited to tweets in the English language. Tweets were also detected in Spanish, Turkish, Japanese, Portuguese, German, Slovenian, and Dutch. However, non-English tweets comprised less than 1% of the total corpus of the initial tweets reviewed. Google Translate was used to translate and interpret non-English tweets.

### Theoretical Framework

The World Health Organization’s (WHO) Strategic Advisory Group of Experts (SAGE) Working Group Vaccine Hesitancy Matrix was the underlying theoretical framework for data coding and classification [14]. This framework includes groupings for contextual influences, individual and group influences, and vaccine or vaccination-specific issues, including those specific to misinformation and disinformation. In this study, we were interested in user-propagated misinformation that fell under contextual influences and a SAGE subcategory entitled “Influential leaders, immunization program gatekeepers, and anti- or pro-vaccination lobbies.” Other data labels included news media and public reactions, which fell under contextual influences that were split from the subcategory entitled “Communication and media environment.”

### Content Coding

The Initial Sample of tweets were manually coded by authors AC, MH, and a team member no longer with the research team as (1) reporting or news media, (2) public reaction, or (3) misinformation, if they attempted to link the death of Hank Aaron with administration of the Moderna COVID-19 vaccine. All Twitter comments (direct or quote tweets) originating from misinformation-labeled content were then further classified and coded for purposes of content analysis using an inductive coding scheme based on the WHO SAGE Vaccine Hesitancy Matrix [14]. To minimize any potential repetition between the parent tweet and associated comment tweets, we conducted additional data filtering to identify direct replies and quote tweets that were exact matches, which comprised of only 2 instances in the entire corpus. To minimize loss of context between the parent and comment tweets, we nested all comments under its respective parent tweet for the purposes of content coding and contextual relevance. All tweets included in the subset of misinformation-labeled and SAGE-coded tweets were assessed for user sentiment (eg, negative [disagree], neutral, or positive [agree] sentiment) via manual annotation. From the Initial Sample, we also identified nonmedia accounts in the corpus, which were “Verified” by Twitter (“Verified Account Sample”), and reviewed historical posts from their Twitter timelines occurring after March 2020 (when COVID-19 was declared a national emergency in the United States) to assess for the presence of additional COVID-19 vaccine misinformation content or SAGE-relevant themes, a methodology similar to what was used in a prior COVID-19 vaccine misinformation study [7].

**Figure 1:**
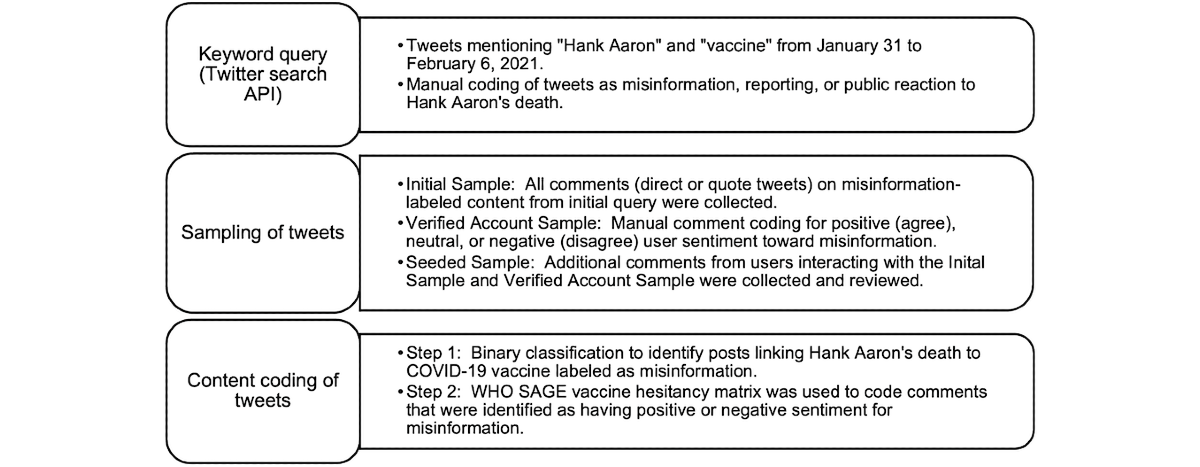
Twitter sampling and coding methodology. API: Application Programming Interface; SAGE: Strategic Advisory Group of Experts; WHO: World Health Organization.

Finally, after manually confirming misinformation or SAGE-related content in the Initial Sample and Verified Account Sample, an additional set of comments from Twitter users interacting with this misinformation content was collected and reviewed for additional misinformation and hesitancy themes (ie, “Seeded Sample”), similar to a snowball sampling approach. Content coding for the Seeded Sample was conducted in August 2021 by author MH and a team member no longer with the research team. Both coders had previous experience and prior publications involving annotation of COVID-19 misinformation-related data and achieved a high intercoder reliability (kappa=0.83) for misinformation codes with discrepancies reviewed and reconciled by rereviewing SAGE categories with all authors [8,15]. A summary of the Twitter sampling and coding methodology is available in Figure 1. A list of the sample positive, neutral, and negative lexicons and terms used specifically for the purposes of manual annotation for sentiment analysis is available in Textbox 1.

**List of representative positive, neutral, and negative lexicons and terms.**

**Positive lexicon and terms (agree)**

- “Not a coincidence” or “Not isolated”
- “Wake up”
- “No vaccine” (Action) or “I will not [get vaccinated]”
- “Exploited”

**Neutral lexicon and terms**

- “So sorry”
- “RIP”
- “[@ mention] Did you see this [The Event]?”
- “<3”

**Negative lexicon and terms (disagree)**

- “Correlation is not causation”
- “Don’t rush”
- “Shame on you”
- “Natural causes”

### Ethical Considerations

In an increasingly digital and globalized world, it is important to consider the challenges that arise when considering user consent, privacy, and social media data use [16]. There does not appear to be clear consensus regarding the proper use of these data, particularly important when assessing the impact of content that may target certain minority populations at higher risk for poorer health outcomes or who may already be underresourced in relation to digital literacy and access to high quality information sources or may lack access to equitable health care services or vaccine coverage [17,18]. It also applies to the analysis of verified accounts with user celebrities and public figures that may actively disseminate questionable information to large numbers of followers. These verified users may have a disproportionate impact on communication dynamics but are not specifically named in this study due to anonymization of results. The utility of infodemiology-informed approaches to rapidly assess emerging public health concerns, such as the infodemic and its associated misinformation-related events, should not minimize these ethical considerations [19]. Hence, one objective of this study was to identify content-specific misinformation themes that may be relevant to a specific population of users but not to overgeneralize findings beyond the exploratory nature of this study and the specific event characterized. We believe results from studies that examine sensitive topics impacting minority health on social media platforms need careful contextualization, otherwise they may risk further eroding public trust among groups already hesitant to engage with biomedical, informatics, and social science researchers.

All information collected from this study was secondary data in the public domain, and the study did not involve any interaction with users. Any user-identifiable information was removed from the study results.

### Funding

Author TKM was supported by funding from the US Food and Drug Administration Office of Minority Health and Equity contract number 75F401120C00181.

## Results

**Figure 2:**
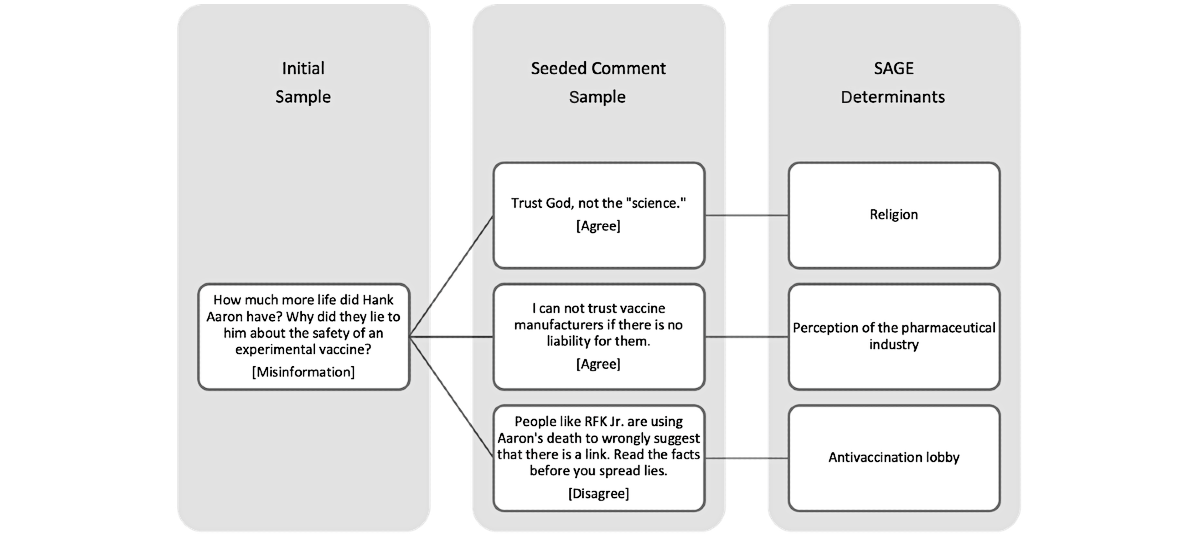
Deidentified example of misinformation spread and impact on vaccine confidence using Strategic Advisory Group of Experts (SAGE) determinants.

A total of 436 tweets were returned from the Twitter Search API based on our keyword query that made up our Initial Sample of tweets collected for this study. After a first round of manual coding for misinformation content per the SAGE categories, vaccine misinformation was found to be the most prominent content type detected (n=244, 56%), followed by public reaction about Hank Aaron’s death from users that did not include misinformation content (n=122, 28%), and media reporting of the event itself (n=69, 16%). Twitter user accounts disseminating or interacting with misinformation-labeled content included verified users with large numbers of followers relative to the entire group of users in the sample (25,000-200,000). Using publicly available profile metadata (ie, user account descriptions and biographies), several verified Black celebrities with large follower counts, including a singer and songwriter, a former National Basketball Association player, a current MLB player, and a former Congressional candidate, were identified as disseminating or interacting with misinformation-labeled content or had a history of posting COVID-19 vaccine-related misinformation and SAGE-identified hesitancy themes during the COVID-19 pandemic (Table 1; deidentified tweet examples and interactions are shown in Figure 2). The majority of users in our Initial Sample were nonverified and had much lower follower counts (mean=2539, max=87,505, min=0) compared with the Verified Account Sample. Notably, none of the misinformation-labeled content reviewed at the time of our content analysis in the Initial Sample or Verified Account Sample was labeled as misleading by Twitter as defined by the platform’s COVID-19 misleading information policy. However, some content has been removed or deleted since initial data collection.

An additional 1243 comments, mostly consisted of quote tweets (n=852, 68.5%) from 973 unique users interacting with misinformation-labeled content, were then collected in the Seeded Sample, with 779 total comments included for analysis after determining that they had some degree of positive or negative sentiment toward misinformation. The majority of these comments expressed positive sentiment or agreement (n=612, 78.6%) with misinformation-labeled content and did not refute it. Common misinformation themes observed included linking the death of Hank Aaron to “suspicious” elderly deaths following vaccination, misinterpretations of VAERS data, claims that federal officials were targeting Black Americans, and other widely espoused and debunked COVID-19–related conspiracies such as depopulation, death panels, and mainstream media collusion with pharmaceutical companies and billionaires such as Bill Gates having sinister motives.

**Table 1:** Examples of deidentified misinformation content and Strategic Advisory Group of Experts vaccine hesitancy themes from verified Twitter users.

Occupation	Follower count, n	Vaccine misinformation tweets detected during COVID-19 (n)	Vaccine misinformation content	WHO^a^ SAGE^b^ vaccine hesitancy themes
Singer-songwriter	25,000-200,000	Yes (<5)	Doctor’s suspicious death after vaccine, concern about vaccine ingredients	Perception of the pharmaceutical industry
Current MLB^c^ player	25,000-200,000	Yes (<5)	Bio-encoded vaccination history placed in the body, Hank Aaron was targeted, vaccines are forced upon less educated communities, agreeing with comments from Del Bigtree, a known anti-vaxxer.	Vaccine development, historical influences, risk outweighs benefit
Former NBA^d^ player	25,000-200,000	Yes (>5)	DNA replacement, engineered SARS-CoV-2 mutants, sterilization, Satanism, depopulation, tracking chips, Bill Gates, Anthony Fauci, Hank Aaron	Vaccine safety, media environment, historical influences, politics
Former Congressional candidate	25,000-200,000	Yes (>5)	Rushed development, celebrities receive “safe” vaccines, misrepresented survival rate, supporting misinformation from Rep. Marjorie Taylor Green, questioning death of Earl Simmons (aka DMX)	Vaccine development, historical influences, risk outweighs benefit, media environment

^a^WHO: World Health Organization.

^b^SAGE: Strategic Advisory Group of Experts.

^c^MLB: Major League Baseball.

^d^NBA: National Basketball Association.

Based on the SAGE categories for vaccine hesitancy in these user reactions to our Initial Sample of misinformation posts, 12% (n=93) of comments mentioned contextual influences (eg, sociocultural, historic, and media environment), 65% (n=508) mentioned individual and group influences (eg, personal or peer perceptions of vaccine), and 14% (n=110) mentioned vaccine-specific issues (eg, concerns about mode of administration). Only 9% (n=70) of comments were not classified within the SAGE misinformation or hesitancy categories. Deidentified examples of identified SAGE categories for each of the above categories are provided in Table 2. Additionally, Figure 2, illustrates the interaction components between misinformation occurring in the Initial Sample, user agreement or disagreement in the Seeded Sample, and the corresponding SAGE vaccine misinformation or hesitancy category.

**Table 2:** Deidentified and paraphrased examples of tweets with detected Strategic Advisory Group of Experts themes.

Category and determinants			Example tweets (paraphrased)
**Contextual**			
	Communication and media environment	You already do not see real information in the media. How can we address this? I am tired of not seeing the real information.	
	Historical influences	It‘s okay to be hesitant. Tuskegee, thalidomide, and the 1976 swine flu vaccine. Old vaccine manufacturers were sued, but COVID-19 vaccine manufacturers have no liability.	
	Politics	Vaccinated and dead two weeks later, but Biden supporters will tell you he was just old.	
**Individual or group**			
	Risk or benefit	Hank Aaron got the [COVID-19] vaccine. It is too risky to receive it right now.	
	Providers’ trust	Doctors who say no one dies from taking the [COVID-19] vaccine are lying. They never tell the truth.	
	Immunization is not needed	Give an alternative to the vaccine like vitamins and zinc.	
**Vaccine or vaccination-specific**			
	Attitude of health care professionals	They are getting everyone to get Black people to vote, even Black doctors to help convince Black people to get vaccinated. Insulting. Culture, not racism, is the issue for Black people.	
	Introduction of a new vaccine	Vaccines help prevent disease, but can still be dangerous, especially if they are new.	
	Design of vaccination program	This vaccine was rushed and was not texted for years. They should not do vaccine mandates, otherwise this will be a problem.	

## Discussion

### Principal Findings

Understanding the drivers of vaccine hesitancy in certain demographic and minority groups will be crucial in stopping the spread of the COVID-19 pandemic, especially as the world experiences vaccine inequity and variant-specific surges that disproportionately impact certain populations. Vaccine hesitancy is complex, influenced by contextual, individual and group-level, and vaccine or vaccination-specific factors that may be specific to minority groups or communities [20]. In this study, we observed a high proportion of interaction with COVID-19 misinformation specific to Black Americans that could unduly influence vaccine confidence and increase hesitancy in this heterogenous community. The untimely death of Hank Aaron, a celebrated Black athlete who expressed his public support for vaccination, was instead appropriated by antivaccination groups to spread misinformation. We observed that antivaccination actors quickly seized on the news of Hank Aaron’s death to advance dubious claims questioning the safety of COVID-19 vaccines. We also observed several celebrities from the Black community participating in misinformation dissemination before and during the events described in this study.

The WHO has named vaccine hesitancy and the COVID-19 infodemic as a critical global health issue, as it threatens to undermine one of the most important public health tools that can curb rising cases amid the spread of concerning variants, including among disproportionately impacted minority populations [21]. The US Surgeon General has also called attention to rampant COVID-19 misinformation on social media platforms, which may further contribute to vaccine hesitancy and lack of uptake (including for boosters), especially in communities with a high Centers for Disease Control and Prevention social vulnerability index, where individuals may be at higher risk of COVID-19 incidence, hospitalization, morbidity, and mortality [22,23]. It is important to recognize that misinformation from antivaccination groups has the potential to compound medical mistrust and vaccine hesitancy by advancing false narratives based on erroneous and misleading information [24,25].

Our study detected high activity of dissemination of misinformation associated with Hank Aaron’s death primarily originating from well-known antivaccination individuals, which were not labeled by Twitter as “misleading” at the time of review. By not labeling this content, this may have allowed for rumors around the death of Hank Aaron to be shared and modified by users concerned about the reasons for his death and may have also seeded further misinformation and conspiracies concerning deaths of other prominent Black Americans or public figures. This study ultimately found that misinformation compounded individual and community-level concerns about COVID-19 vaccines at a time during their crucial early adoption. These concerns were more prominent within the social media discourse surrounding Hank Aaron’s death compared to the contextual-level (eg, media environment) or vaccination-specific concerns (eg, adverse effects). This suggests that social media and other online forums may not be the opportune venue to promote vaccine confidence and debunk misinformation unless health promotion is targeted and contextualized to the attitudes, beliefs, and unique contextual factors of different user groups [26].

In fact, Twitter accounts from prominent well-known antivaxxer personalities, where some of the misinformation-labeled content originated in this study, remain active at the time of writing, though some US policy makers have called for accounts from the so-called “Disinformation Dozen” to be suspended from major social media platforms [27,28]. Our results also align with prior studies that have examined COVID-19 vaccine-related misinformation and found that user engagement with this content can have a direct impact on vaccine confidence, highlighting the need for stronger misinformation-specific content moderation policies coupled with more robust and consistent enforcement of new and existing policies [29,30].

### Limitations

There are several limitations to consider in this study. First, in the data collection phase, we requested a maximum of 1000 tweets from January 31, 2021 (start: 9 days after Hank Aaron’s death) to February 6, 2021 (end: 16 days after Hank Aaron’s death), using the Twitter Search API. This query with a narrow, but specific set of keywords returned a relevant sample of 436 tweets, but the use of additional keywords (eg, MLB, baseball player) or a longer data collection period may have yielded additional tweets that may have been different for the purposes of content analysis. Hence, the results of our study are likely not generalizable to this specific infodemic event. Instead, the objective of this study was to generate an initial sample of tweets directly relevant to Hank Aaron’s untimely passing and then obtain a larger sample of user comments, interactions, and dissemination behavior by digitally “snowballing” this initial sample into additional Twitter user-generated discussions. This approach may be useful for in-depth characterization of narrow event-driven infodemic detection but has limited generalizability compared to larger scale studies that employ approaches using topic modeling, natural language processing, and supervised machine learning. Additionally, our use of follower counts in the Verified Account Sample has limitations associated with its ability to characterize misinformation dissemination as it may not reflect users’ actual activities. A full social network analysis of our data set may have better elucidated communication structures (eg, information flow) and active user groups but was beyond the scope of this study [31,32]. Specifically, this analysis might have identified influential users in the Hank Aaron misinformation discourse, though for this study, we chose to focus on user sentiment to provide a more in-depth characterization of misinformation themes given the generally small size of the overall study data set (ie, both the Initial Sample and Seeded Sample). Future event-driven studies should explore the use of social network analysis and active audience analysis to measure user influence and message diffusion more robustly on social media platforms, particularly with viral misinformation content [33].

### Conclusion

Close to half a year after his passing, we continued to observe misinformation surrounding Hank Aaron’s death propagating on social media networks. Other deaths of prominent African American people (eg, rapper Earl Simmons, “DMX”) have also been erroneously connected with the COVID-19 vaccine by similar antivaccination groups. Hence, the real-world impact of misinformation on vaccine confidence and hesitancy in minority communities, both online and offline, needs to be addressed urgently, as the legacy of changemakers such as Hank Aaron should be about his accomplishments on and off the field, not a field of misinformation.

## Abbreviations

API: Application Programming Interface
MLB: Major League Baseball
SAGE: Strategic Advisory Group of Experts
VAERS: Vaccine Adverse Event Reporting System
WHO: World Health Organization

## Data Availability

Deidentified data with applicable Twitter identifications are available from authors upon request.
